# Multimodal molecular representation and property prediction based on structured complementary feature fusion

**DOI:** 10.3389/fphar.2026.1844064

**Published:** 2026-09-30

**Authors:** Meng Ma, Qingqing Cao, Fang Liu, Caihua Chen, Yan Liu

**Affiliations:** College of Information Engineering, Hunan Open University, Changsha, China

**Keywords:** conformational consistency refinement, molecular property prediction, molecular representation, multi-modal representation learning, self-supervised learning

## Abstract

In this work, we propose Molrep-SCF, a multimodal self-supervised pretraining framework for molecular representation learning using SMILES sequences, 2D molecular graphs, and 3D geometric structures. The three modalities are encoded using a Transformer, a graph neural network, and an invariant 3D graph neural network, respectively. To enrich geometric supervision beyond a single conformation, Molrep-SCF incorporates three selected and complementary 3D conformational views. We further introduce a 2D-guided conformational consistency refinement mechanism that uses molecular topology to guide the fusion and refinement of their geometric representations. Instead of relying on a single static conformation, our method enforces structural consistency across conformers under the guidance of 2D topological priors. Furthermore, we design a structure-aware cross-modal learning strategy with atomic-level alignment via position-specific masking, enabling fine-grained correspondence across modalities. In addition, masked reconstruction across modalities enhances deep semantic coupling and facilitates information exchange between heterogeneous representations. Extensive experiments demonstrate that Molrep-SCF consistently outperforms existing multi-modal frameworks on molecular property prediction and other tasks, showing stronger generalization and representation quality.

## 1 Introduction

Molecular representation learning serves as the fundamental cornerstone of cheminformatics and Computer-Aided Drug Design (CADD) (Niazi and Mariam, 2023; Suryanarayanan et al., 2026). Accurate molecular representations establish a vital bridge between microscopic structures and macroscopic biological activities or physicochemical properties, thereby playing a decisive role in critical tasks such as virtual screening, toxicity assessment, and materials design (Hassan Baig et al., 2016; Zhang et al., 2024a).

However, in practical applications, acquiring high-quality experimental labels for molecular data is often hindered by prohibitive costs and long experimental cycles. To overcome the scarcity of labeled data, research has increasingly shifted toward self-supervised learning (SSL) (Jaiswal et al., 2020; Wei et al., 2026). By constructing pretext tasks or pseudo-labels from the internal structure of unlabeled data, SSL enables models to capture intrinsic structural features and contextual information, facilitating effective transfer learning for downstream tasks.

In the field of molecular modeling, various SSL paradigms have been developed to mine intrinsic molecular features. These include graph neural networks (GNN) combined with masked modeling, such as MOLE-BERT (Xia et al., 2023) and MoLGNN (Shen et al., 2020), as well as pre-training methods enhanced by score-based denoising (Ni et al., 2024); sequence-based language models, including ChemBERTa-2 (Ahmad et al., 2022), CSGL (Li et al., 2025), and TopoFormer (Chen et al., 2024); methods incorporating geometric information and structural relationships, such as geometry-enhanced representation learning (Fang et al., 2022), Transformers for protein-ligand interaction prediction (Pourmirzaei et al., 2025), and chemical synthesis graph learning (Xie et al., 2024); and contrastive learning frameworks, ranging from attention-guided positive sample selection (Forooghi et al., 2026) to strategies addressing negative sample mitigation and fragment-level contrastive improvements (Tang et al., 2025). By leveraging contrastive learning or generative models—such as Variational Autoencoders (VAEs) (Diederik and Max, 2019) or Generative Adversarial Networks (GANs) (Goodfellow et al., 2020)—these methods extract key structural features from massive unlabeled compound datasets, which is particularly advantageous for property prediction tasks where labeled data is extremely limited.

As multi-modal research advances, increasing attention has been directed toward the synergistic representation of molecular modalities. Recently, frameworks such as MolPROP (Rollins et al., 2024), MolMVC (Huang et al., 2024), MolFeSCue (Zhang et al., 2024b) have emerged. These frameworks represent molecular SMILES, 2D graphs, 3D structures, and textual descriptions as distinct modalities, further leveraging self-supervised learning strategies for pre-training to facilitate various downstream tasks. While significant progress has been achieved, challenges remain in this domain (Figure 1). Firstly, molecules may adopt multiple conformations, and representing a molecule using only the lowest-energy or a single fixed conformation may provide limited geometric information. Secondly, most existing methods focus on capturing shared information between modalities but lack deep modeling of the semantic coupling relationships, leading to the potential loss of critical information. Finally, existing 3D molecular modeling approaches are often sensitive to conformational variations, leading to inconsistent molecular representations. Although MolMix (Manolache et al., 2024) explores the integration of multiple conformers, particularly for flexible molecules, it primarily aggregates conformer-level representations without explicitly modeling topology-guided interactions among the selected conformational views. MolFusion (Cai et al., 2025) introduces atom-level masking and cross-modal alignment between SMILES and 2D graphs, yet it does not extend such structured alignment to 3D conformations or jointly coordinate the semantic relationships among 1D, 2D, and 3D modalities.

**FIGURE 1:**
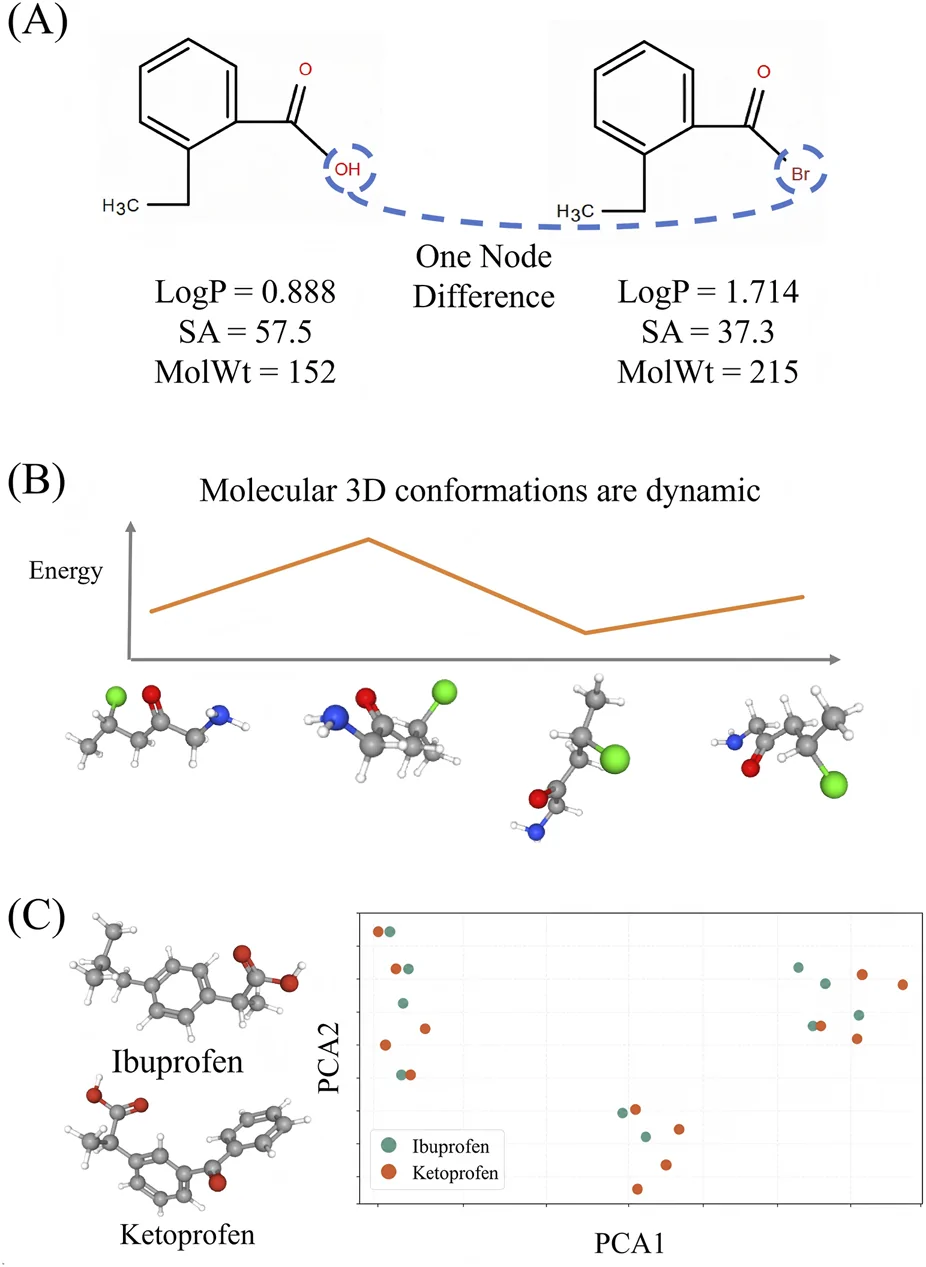
Motivations. **(A)** Two molecules may differ by only one atom, yet exhibit vastly different properties. **(B)** A molecule may adopt multiple conformations, resulting in geometric variability. **(C)** The PCA visualization shows that different conformers of the same molecule exhibit substantial embedding variability, indicating instability in 3D conformational representations (Embeddings from DimeNet (Gasteiger et al., 2020)).

To address the issues, we propose Molrep-SCF, a unified multi-modal pre-training framework based on structured complementary feature fusion. Our model first acquires the SMILES, 2D graphs, and 3D structures of a molecule, encoding them using Transformer, GNN, and Invariant 3D GNN respectively. Prior to encoding, we implement a sophisticated masking strategy that processes identical and distinct atoms separately to force the model to learn deep cross-modal structural associations. To address the challenge of dynamic 3D conformational variability, we propose a 2D-guided 3D Consistency Refinement mechanism. Instead of relying on probabilistic latent modeling, we promote representational consistency among several selected 3D conformational views by leveraging 2D topological guidance. The primary contributions of this work are summarized as follows:

- Atomic-level Alignment via Position-specific Masking: We design a precise alignment mechanism that masks atoms at the same positions across different modalities. This forces the model to pull these representations closer in the feature space, ensuring consistency across 1D, 2D, and 3D modalities and enhancing the capture of shared multi-modal features.
- Structure-driven Cross-modal Semantic Coupling: To resolve the coupling deficiency, we employ a strategy that masks distinct molecular fragments. By requiring the model to reconstruct masked content in one modality using visible information from another, we achieve effective modeling of complex, deep-seated semantic relationships across modalities.
- 2D-Guided Conformational Consistency Refinement: We propose a 2D-guided conformational consistency learning mechanism to address the variability of molecular 3D structures. Instead of relying on a single conformer or simple averaging across multiple conformations, our method explicitly leverages the 2D molecular topology as a structural prior to guide the aggregation and refinement of selected 3D conformational views.

## 2 Methods

### 2.1 Data preparation

To ensure the model learns generalized molecular representations across modalities, this study encompasses a complete data pipeline from large-scale unsupervised pre-training to multi-scenario downstream task fine-tuning. We utilize the PCQM4Mv2 dataset (Lu et al., 2024) (from the OGB large-scale benchmark) for multi-modal pre-training, which contains 3.37 million molecules with SMILES sequences, 2D molecular graphs, and 3D geometric structures.

During the data preprocessing stage, we perform several specific operations:

- Element Filtering: The BPE (Byte Pair Encoding) vocabulary used in this model is statistically generated from the ChEMBL database SMILES corpus. Since this vocabulary does not include Germanium (Ge), we remove all molecules containing Ge from the PCQM4Mv2 dataset to ensure encoding consistency.
- 3D Augmentation: In addition to the original 3D coordinates provided by PCQM4Mv2, we generate multiple candidate conformers using the RDKit toolkit and optimize them with the MMFF94 force field. The conformers are ranked according to their MMFF94 energies, and the first- and fifth-lowest-energy conformers among the five lowest-energy candidates are selected as two additional 3D inputs. Together with the original structure, these conformers provide three complementary geometric views that capture both stable and moderately varied molecular configurations.

To evaluate generalization, the dataset is divided into training, validation, and test sets with a ratio of 98:1:1. All baseline models are trained and evaluated under the same experimental settings to ensure a fair comparison. The overall workflow of the proposed framework is illustrated in Figure 2, encompassing multi-modal encoding, synergistic alignment, and structure-driven deep fusion.

**FIGURE 2:**
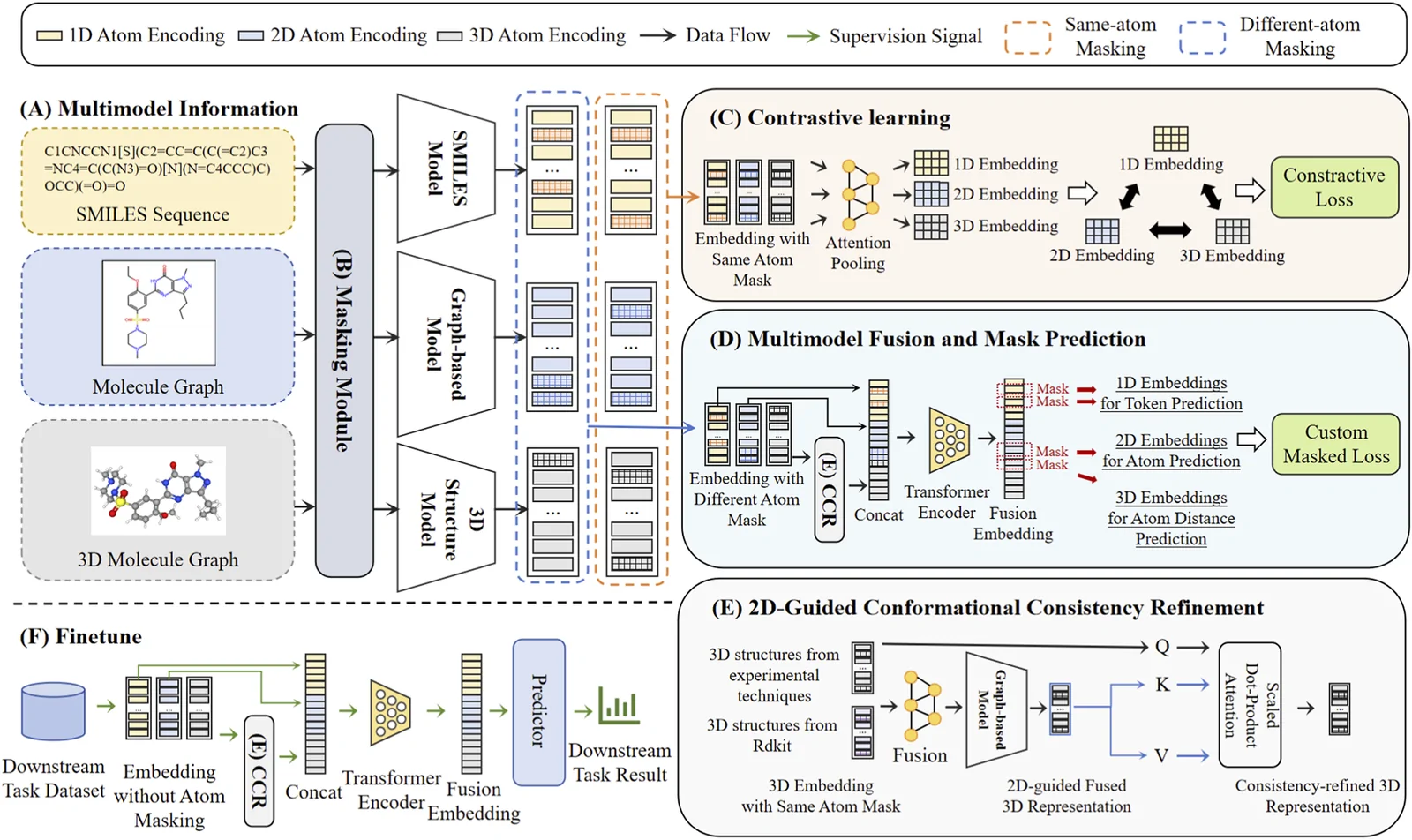
Overview of Molrep-SCF. **(A)** Input molecular data, including SMILES sequences, molecule graphs, and 3D molecular structures, are processed for feature encoding. **(B)** Different masking strategies. **(C)** Embeddings from the same atom positions across different modalities are aligned using attention pooling, minimizing contrastive loss to improve representation consistency. **(D)** Embeddings from multiple modalities are fused, and decoding strategies are employed to predict masked atoms and atom positions. **(E)** Illustration of the proposed 2D-guided conformational consistency refinement module, where three selected 3D conformational views are fused and refined under 2D structural guidance under 2D structural guidance, followed by attention-based interaction to produce the final 3D representation. **(F)** Fine-tuning is performed using downstream task datasets.

### 2.2 Molecular multi-modal encoding

This framework designs three parallel encoding branches to capture chemical features from different dimensions: 1D SMILES sequences, 2D topological graphs, and 3D geometric structures.

#### 2.2.1 SMILES-based 1D sequence encoding

For any molecule q , we treat its SMILES not merely as a character sequence but as a carrier of chemically meaningful substructure semantics. We use the Explainable Substructure Partition Framework (ESPF) (Huang et al., 2019) to identify key functional groups and divide the SMILES into a sequence of e segments based on a substructure vocabulary V , denoted as $E_{q}=\left\{S_{1},\ldots ,S_{e}\right\}$ , where $S_{i}\in V$ . Each token is mapped into a continuous representation via an embedding layeras shown in Equation 1:

$$X_{q}=\text{Embed}\left(E_{q}\right).$$ (1)

To incorporate positional information, we introduce learnable positional embeddings $P\in R^{e\times d}$ , and obtain the input representation as shown in Equation 2:

$$h_{q}=X_{q}+P.$$ (2)

A Transformer encoder is employed to perform context mining on the ESPF sequence. It consists of stacked blocks containing a multi-head attention layer and a feed-forward layer to capture long-range chemical dependencies within the segment sequence as shown in Equation 3:

$$h_{q}^{1\text{D}}=\text{LayerNorm}\left(h_{q}+\text{FeedForward}\left(\text{MultiHeadAttention}\left(h_{q}\right)\right)\right)$$ (3)

#### 2.2.2 Graph-based 2D topology encoding

We represent the molecule as a graph G=(N,E) , where nodes N represent atoms and edges E represent chemical bonds. To capture subtle topological variations, such as the specific positioning of functional groups, we use a Graph Isomorphism Network (GIN) (Wang and Zhang, 2022) as the 2D encoder. In each iteration k , the feature vector $g_{v}$ of atom node v evolves by aggregating its neighborhood features as shown in Equation 4:

$$g_{v}^{\left(k\right)}={\text{MLP}}^{\left(k\right)}\left(\left(1+\epsilon^{\left(k\right)}\right)\cdot g_{v}^{\left(k-1\right)}+\underset{u\in N\left(v\right)}{\sum }g_{u}^{\left(k-1\right)}\right)$$ (4)

where N(v) is the set of neighbors of atom v , $\epsilon^{\left(k\right)}$ is a learnable parameter, and ${\text{MLP}}^{\left(k\right)}$ denotes a multi-layer perceptron for non-linear mapping. The final node-level representation for molecule q is denoted as $h_{q}^{2D}\in R^{n\times d}$ , where n is the number of atoms and d is the hidden dimension.

#### 2.2.3 Multi-conformer 3D geometric encoding

We adopt a Directional Message Passing Neural Network (DimeNet) (Gasteiger et al., 2020) as the 3D geometric encoder. Unlike conventional GNNs that rely only on pairwise distances, DimeNet incorporates angular information by modeling triplet interactions between atoms.

For each directed edge (j,i) , the edge representation is updated via directional message passing as shown in Equation 5:

$$m_{ji}^{\left(l+1\right)}=\text{MLP}\left(m_{ji}^{\left(l\right)}+\underset{k\in N\left(j\right)\backslash i}{\sum }\Phi \left(m_{kj}^{\left(l\right)},\;e_{ji},\;a_{\mathrm{kji}}\right)\right),$$ (5)

where $e_{ji}$ denotes radial basis function (RBF) embeddings of interatomic distances, and $a_{\mathrm{kji}}$ represents angular basis functions capturing bond-angle interactions.

The angular information is encoded using spherical basis functions constructed from spherical Bessel functions and real spherical harmonics as shown in Equation 6:

$$a_{\mathrm{kji}}=\text{SBF }\left(d_{kj},\theta_{\mathrm{kji}}\right)={\left\{j_{l}\left(\frac{z_{ln}}{c}d_{kj}\right)Y_{l}^{0}\left(\theta_{\mathrm{kji}}\right)\right\}}_{l,n},$$ (6)

where c is the cutoff radius and $z_{ln}$ denotes the n -th root of the l -th spherical Bessel function. The final 3D atomic representation is obtained by aggregating directional messages after L interaction blocks as shown in Equation 7:

$$h_{i}^{3D}=\text{Readout}\left(\left\{m_{ji}^{\left(L\right)}|j\in N\left(i\right)\right\}\right),$$ (7)

yielding the resulting molecule-level representation is $h_{q}^{3D}\in R^{n\times d}$ .

### 2.3 Cross-modal synergistic semantic consistency alignment

To capture the deep semantic coupling relationships of molecules across different modalities and obtain shared feature information, this framework designs a cross-modal synergistic alignment strategy. This strategy employs a co-masking mechanism and contrastive learning to force the model to align 1D, 2D, and 3D features within a unified latent space, thereby achieving the complementarity and integration of multi-dimensional information. During the pre-training stage, if an atom or substructure in one modality (e.g., 1D SMILES) is masked, the corresponding atom nodes in the 2D topological graph and 3D geometric structure will be synchronously masked.

As shown in Figure 2C, the masked representations processed by their respective encoders are aggregated through an Attention Pooling layer. By learning the weights of different atoms or fragments, this layer compresses variable-length sequence or graph node features into fixed-dimensional global modal embedding vectors: $H_{1D}$ captures the chemical substructure semantics in the sequence, $H_{2D}$ captures the topological connection relationships of the molecule, and $H_{3D}$ captures complementary spatial features from the selected conformational views.

To pull the different modal representations of the same molecule closer in the latent space while pushing those of different molecules apart, we employ three pairs of cross-aligned InfoNCE losses Parulekar et al. (2023). InfoNCE aims to maximize the mutual information between positive sample pairs through contrastive learning. For a given pair of modal embedding vectors $\left(H_{x},H_{y}\right)$ , the loss function in a single direction is defined as as shown in Equation 8:

$$\ell \left(H_{x},H_{y}\right)=-\log\frac{\exp\left(\text{sim}\left(H_{x},H_{y}\right)/\tau \right)}{\sum_{k=1}^{B}\exp\left(\text{sim}\left(H_{x},H_{k}\right)/\tau \right)}$$ (8)

where sim(⋅,⋅) denotes cosine similarity, B is the current batch size, and τ is the temperature hyperparameter used to adjust the concentration of the similarity distribution. The total contrastive loss $L_{\mathrm{con}}$ is composed of three pairwise alignment terms as shown in Equation 9:

$$L_{\mathrm{con}}=\ell \left(H_{1D},H_{2D}\right)+\ell \left(H_{2D},H_{3D}\right)+\ell \left(H_{1D},H_{3D}\right)$$ (9)

This tri-modal cyclic alignment architecture ensures that 1D–2D–3D information is jointly learned during the pre-training stage. This effectively avoids representation bias from any single modality and significantly enhances the model’s capability to model complex molecular properties.

### 2.4 Structure-driven cross-modal deep fusion

To address the limitations of traditional multi-modal methods that focus solely on embedding space alignment while lacking deep semantic coupling, this framework proposes a structure-driven deep fusion mechanism. By employing a complementary masking strategy and a shared feature evolution process, the model captures complex high-order structural relationships and non-linear cross-modal dependencies.

As illustrated in Figure 2D, a Different Atom Masking strategy is introduced during the deep fusion stage, which differs from the strategy used in the alignment phase. Under this mechanism, if the i -th atom is masked in one modality (e.g., 1D SMILES), the corresponding i -th atom in the 2D topological graph and 3D geometric representation must remain visible. This non-overlapping masking design forces the model to utilize cross-modal complementary information—such as 2D neighborhood topology and 3D spatial geometry—to infer missing 1D tokens, thereby achieving deep semantic coupling and information flow at the feature level.

To further enhance cross-modal geometric consistency, we introduce a 2D-guided conformational consistency refinement (CCR) mechanism at the 3D representation level, as shown in Figure 2E. Unlike conventional approaches that directly aggregate several conformational views, our method uses 2D topology to guide the refinement of the fused representations obtained from the three selected views.

For each molecule, we use three selected conformational views, consisting of the original structure and two MMFF94-ranked conformers. Their atom-level representations are denoted as ${\left\{h^{3D,\left(m\right)}\right\}}_{m=1}^{3}$ . We perform deterministic feature fusion by concatenation followed by linear projection as shown in Equation 10:

$$h_{\mathrm{fuse}}^{3D}=W_{f}\left[h^{3D,\left(1\right)}\|h^{3D,\left(2\right)}\|h^{3D,\left(3\right)}\right],$$ (10)

where ‖ denotes feature concatenation.

To inject geometric information into the molecular topology, the fused 3D features are used as node inputs to the 2D GNN as shown in Equation 11:

$$h^{2D^{\prime }}={\text{GNN}}_{2D}\left(h_{\mathrm{fuse}}^{3D},E\right),$$ (11)

where 3D features serve as additional node-level geometric priors.

Finally, we perform cross-modal attention from 2D-to-3D for conformational refinement. Given 2D node embeddings $h^{2D^{\prime }}$ and original 3D representations $h_{3D}$ , attention weights are computed as shown in Equation 12:

$$\alpha_{ij}=\frac{\exp\left(\phi {\left(h_{i}^{3D}\right)}^{⊤}\psi \left(h_{j}^{2D^{\prime }}\right)\right)}{\sum_{k\in B\left(i\right)}\exp\left(\phi {\left(h_{i}^{3D}\right)}^{⊤}\psi \left(h_{k}^{2D^{\prime }}\right)\right)}.$$ (12)

where i indexes atoms in the 3D conformations, while j indexes atoms in the 2D graph. The attention operation is restricted to atoms belonging to the same molecule through batch-wise masking. B(i) denotes the set of 2D atoms belonging to the same molecule as the i -th 3D atom.

The refined 3D representation is as shown in Equation 13:

$$H_{3D}^{\prime }=\rho \left(\underset{i}{\sum }\alpha_{ij}h_{i}^{3D}\right),$$ (13)

where ρ(⋅) is a nonlinear projection network with LayerNorm.

This design enables a unidirectional interaction from topology to geometry, where 2D structural priors guide and refine 3D conformational representations, leading to molecular representations that are more stable across the selected conformational views. After this, the masked representations generated by each branch are integrated through a concatenation operation to form a long sequence feature encompassing 1D, 2D, and 3D information as shown in Equation 14:

$$H_{\mathrm{concat}}=H_{1D}\|H_{2D}\|H_{3D}^{\prime }$$ (14)

Subsequently, these concatenated features are fed into a shared Transformer encoder. By leveraging the multi-head self-attention mechanism, the model performs non-linear modeling of structural information across modal boundaries, ultimately outputting a fusion embedding with deeply coupled semantics.

Based on the fusion representation, the model executes three parallel masked prediction tasks through dedicated decoding heads to verify the depth of its structural understanding:

#### 2.4.1 1D Masked Token Prediction $\left(L_{1D}\right)$

A masked cross-entropy loss is used to predict the masked SMILES segment tokens as shown in Equation 15:

$$L_{1D}=-\frac{1}{N_{\mathrm{mask}}}\underset{i\in \text{mask}}{\sum }s_{i}\log\left({\hat{s}}_{i}\right)$$ (15)

where ${\hat{s}}_{i}$ is the predicted probability of the token category. This loss encourages the model to capture the underlying logic of chemical syntax.

#### 2.4.2 2D Structural Symmetry Loss $\left(L_{2D}\right)$

To enhance the discriminative power of graph representations, a Symmetric Cross Entropy (SCE) loss is applied as shown in Equation 16:

$$L_{2D}=\frac{1}{N}\overset{N}{\underset{i=1}{\sum }}{\left(1-\text{sim}\left({\hat{h}}_{2D,i},h_{2D,i}\right)\right)}^{\alpha }$$ (16)

where α is a balancing parameter used to guide the model in accurately characterizing topological details.

#### 2.4.3 3D Geometric Denoising Prediction $\left(L_{3D}\right)$

Gaussian perturbations are added to the atomic coordinates of selected substructures as shown in Equation 17:

$$r_{i}^{\prime }=r_{i}+\epsilon_{i},\;\epsilon_{i}∼N\left(0,\sigma^{2}I\right).$$ (17)

The perturbed conformer is processed by the 3D encoder. Rather than reconstructing absolute Cartesian coordinates, the model predicts the clean pairwise distances between perturbed atoms and their local neighbors as shown in Equation 18:

$$d_{ij}={\left\|r_{i}-r_{j}\right\|}_{2},\;{\hat{d}}_{ij}=g_{\text{dist}}\left(h_{i}^{3D},h_{j}^{3D}\right).$$ (18)

The invariant geometric denoising objective is defined as shown in Equation 19:

$$L_{3D}=\frac{1}{\left|P\right|}\underset{\left(i,j\right)\in P}{\sum }\text{SmoothL}1\;\left({\hat{d}}_{ij},d_{ij}\right),$$ (19)

where P denotes the local atom pairs associated with the perturbed substructures. Since pairwise distances are invariant to global translations and rotations, this objective is consistent with the invariant representations produced by DimeNet.

The total loss for the fusion module is defined as shown in Equation 20:

$$L_{\mathrm{fusion}}=L_{1D}+L_{2D}+L_{3D}$$ (20)

Through this “structure-driven” joint training, the model not only learns single-modality features but also captures latent cross-modal coupling patterns. This significantly enhances the robustness of the representations in downstream tasks.

### 2.5 Total objective

Finally, the total loss function for the pre-training stage is defined as the weighted sum of the losses from the three functional modules as shown in Equation 21:

$$L_{\mathrm{total}}=\lambda_{\mathrm{con}}L_{\mathrm{con}}+\lambda_{\mathrm{fus}}L_{\mathrm{fusion}}$$ (21)

By jointly optimizing these modules, the model learns a unified molecular representation that is highly robust, deeply coupled in semantics, and enriched with complementary multimodal structural and geometric information.

### 2.6 Fine-tuning for downstream tasks

Following the completion of multi-modal pre-training, the model possesses the capability to capture cross-modal consistency and deep semantic coupling within molecular structures. As depicted in Figure 2F, the pre-trained encoders and the fusion module are transferred to downstream tasks for molecular property prediction via supervised learning.

## 3 Results

To assess the versatility of Molrep-SCF, we perform comprehensive experiments with diverse data formats, leveraging four prominent benchmarks: MoleculeNet (Wu et al., 2018) based on 2D molecular data, QM9 (Ramakrishnan et al., 2014) with 12 tasks utilizing 3D molecular data, Drug-Target Binding Affinity (DTA) (Nguyen et al., 2021) and Cellular Drug Response (CDR) (Jiang et al., 2022). The DTA task involves predicting the binding affinity between a drug and its target protein, which is crucial in drug discovery. The CDR task focuses on predicting cellular responses to different drug compounds, providing insights into the effectiveness of these compounds at the cellular level. Additionally, we conduct ablation studies to analyze the contributions of key modules, accompanied by visualization techniques. Furthermore, we provide detailed case studies to highlight the effectiveness of multi-view fusion.

### 3.1 Downstream datasets and evaluation protocols

MoleculeNet consists of eight tasks based on 2D molecular data, covering various domains such as molecular property prediction and molecular activity. These tasks are split based on scaffold partitioning, a method that divides molecules into distinct scaffold groups to ensure that the training, validation, and test sets contain non-overlapping molecular structures. For each task, we report the mean and standard deviation of the results across three random seeds to account for variability in the model’s performance.

QM9, on the other hand, consists of 12 tasks involving 3D molecular data, specifically designed for quantum mechanical properties prediction. In our experiments, we randomly split the 10,000 and 10,831 molecules into validation and test sets, respectively, with the remaining molecules used for fine-tuning.

For the DTA experiments, each sample consists of a drug molecule, a target protein sequence, and the corresponding affinity label. The pretrained Molrep-SCF encoder is used to replace only the original drug encoder, while the target sequence is processed by the protein encoder of the corresponding baseline. For molecules without available 3D structures, the lowest-energy conformer generated using RDKit is used as the 3D input.

For the CDR experiments, the pretrained Molrep-SCF encoder represents the drug molecule, whereas the cell-line features are processed by the original cell-line encoder. The resulting representations are fused and passed to the unchanged prediction head, while all task-specific components other than the drug encoder remain consistent with the corresponding baseline.

### 3.2 Baselines

We select the most representative 2D and 3D pretraining baselines, as well as recently published methods, for our experiments. The selected baselines include MolCLR (Wang et al., 2022), GROVER (Rong et al., 2020), GraphMAE (Hou et al., 2022), CSGL (Li et al., 2025), GraphMVP (Liu et al., 2021), DHTNN (Song et al., 2023), MolPROP (Rollins et al., 2024) and 3D InfoMax (Stärk et al., 2022). For the QM9 dataset, we also include the Distance Prediction (Liu et al., 2022), 3D InfoGraph (Sun et al., 2020) and MoleculeSDE (Liu et al., 2023) for comparison. To ensure a fair comparison, we adopt a unified data splitting strategy across all models and evaluate their performance under consistent conditions, reporting results obtained from identical data partitions and evaluation protocols.

### 3.3 Evaluation of 2D performance

Our experimental setup eliminates the use of 3D structures to guarantee a fair comparison. This decision was made to align with most existing multimodal baseline methods, as these methods do not utilize 3D structures for downstream tasks, mainly because the MoleculeNet dataset does not provide 3D structural data.

Results shown in Table 1. Although 3D structures are not used in downstream tasks, this does not imply that the 3D-aware pretraining signals are ineffective. In our framework, geometric information learned from 3D conformations is implicitly distilled into the 2D representations during pretraining. As a result, the resulting 2D embeddings are enriched with latent 3D structural information, which can still benefit downstream prediction tasks even in the absence of explicit 3D inputs.

**TABLE 1:** Performance comparison of Molrep-SCF with baselines on MoleculeNet.

Methods	BBBP	ToxCast	SIDER	ClinTox	MUV	HIV	Bace	Avg
MolCLR	66.6 ± 1.8	62.9 ± 0.3	57.5 ± 1.7	86.1 ± 0.9	72.5 ± 2.3	76.3 ± 2.4	71.5 ± 3.1	70.48
GROVER	70.0 ± 0.1	65.4 ± 0.4	64.8 ± 0.6	81.2 ± 3.0	67.3 ± 1.6	62.5 ± 0.9	82.6 ± 0.7	70.54
GraphMVP	68.5 ± 0.2	62.7 ± 0.1	62.3 ± 1.6	79.0 ± 2.5	75.0 ± 1.4	74.8 ± 1.4	76.8 ± 1.1	71.30
3D InfoMax	69.1 ± 1.0	64.4 ± 0.8	60.6 ± 0.7	79.9 ± 3.4	74.4 ± 2.4	76.1 ± 1.3	79.7 ± 1.5	72.03
GraphMAE	72.0 ± 0.6	64.1 ± 0.3	60.3 ± 1.1	82.3 ± 1.2	76.3 ± 2.4	77.2 ± 1.0	83.1 ± 0.9	73.61
DHTNN	65.9 ± 0.7	60.4 ± 0.5	59.8 ± 1.7	82.0 ± 1.9	73.4 ± 2.2	76.6 ± 0.4	80.2 ± 2.1	71.19
MolPROP	68.7 ± 0.4	62.9 ± 0.7	60.7 ± 1.2	95.3 ± 1.8	73.5 ± 0.9	76.2 ± 0.9	70.2 ± 1.3	72.50
CSGL	69.5 ± 0.4	66.5 ± 0.6	62.1 ± 1.1	92.3 ± 0.7	74.4 ± 1.7	79.4 ± 2.4	76.1 ± 0.7	74.32
Molrep-SCF	72.8 ± 0.6	67.4 ± 0.7	63.5 ± 0.9	97.5 ± 1.0	76.2 ± 1.2	78.1 ± 1.2	83.2 ± 1.2	76.95

In terms of task performance, our model achieved suboptimal results on the MUV and HIV tasks. While it did not reach the best performance, it still demonstrated strong competitiveness in these tasks. For other tasks, our model performed excellently, especially in the ClinTox task, where it achieved a significant lead over other baseline methods. Overall, our model’s average score is nearly two points higher than that of the second-best model, reflecting its advantage across the entire MoleculeNet dataset. This indicates that our method can maintain efficient predictive performance across different tasks and possesses strong generalization capabilities.

### 3.4 Evaluation of 3D performance

In terms of task performance, for the QM9 dataset, we utilized all three molecular modalities, namely, SMILES, 2D graphs, and 3D geometric structures. The performance comparison in Table 2 shows that Molrep-SCF achieves the best average rank across all tasks, outperforming most baseline methods. Specifically, Molrep-SCF excels in tasks like Alpha, Gap, Mu, Cv, U298, and Zpve, where it consistently delivers the best results. These tasks are critical for modeling molecular properties related to stability and energy, such as Alpha (related to the dipole moment) and Gap (related to the energy gap between the highest occupied molecular orbital (HOMO) and the lowest unoccupied molecular orbital (LUMO)). Molrep-SCF’s strong performance in these areas highlights its capability to capture the electronic structure of molecules effectively.

**TABLE 2:** Performance comparison of Molrep-SCF with baselines on QM9 datasets. The baseline results are taken from the MoleculeSDE.

Methods	Alpha	Gap	HOMO	LUMO	Mu	Cv	G298	H298	R2	U298	U0	Zpve	Avg. rank
Distance prediction	0.065	45.87	27.61	23.34	0.031	0.033	14.83	15.81	0.248	15.07	15.01	1.837	5.750
3D InfoGraph	0.062	45.96	29.29	24.60	0.028	0.030	13.93	13.97	0.133	13.55	13.47	1.644	4.667
3D InfoMax	0.057	42.09	25.90	21.60	0.028	0.030	13.73	13.62	0.141	13.81	13.30	1.670	4.250
GraphMVP	0.056	41.99	25.75	21.58	0.027	0.029	13.43	13.31	0.136	13.03	13.07	1.609	3.000
MoleculeSDE	0.054	41.77	25.74	21.41	0.026	0.028	13.07	12.05	0.151	12.54	12.04	1.580	2.167
Molrep-SCF	0.053	34.52	23.68	21.47	0.023	0.027	13.03	11.87	0.135	11.98	11.65	1.522	1.167

In terms of specific tasks related to molecular stability and thermodynamic properties, G298 and H298 focus on the molecular Gibbs free energy at 298 K and enthalpy at 298 K, respectively, which are essential for understanding molecular stability and reactivity in practical conditions. Molrep-SCF performs exceptionally well in these tasks, outperforming baselines like MoleculeSDE. Furthermore, Molrep-SCF achieves strong results in R2 and U298, which are linked to the molecular prediction accuracy and energy stability at different temperatures. These results demonstrate the model’s robustness and its ability to generalize across various molecular properties, further proving its advantage in tasks that require accurate thermodynamic and electronic property predictions.

### 3.5 Ablation study

In this study, we conduct ablation experiments to assess the contribution of various components of our model. We examine five ablation strategies: (1) No Pretrain, (2) No CCR, (3) No Custom Masked Loss, (4) Random Mask, and (5) Only Contrastive Learning with Random Mask. The last setting retains the same 1D, 2D, and 3D encoders, but replaces the proposed structured fusion and refinement mechanisms with simple concatenation of the multimodal representations. It is trained using only the contrastive objective with random masking, thereby serving as a parameter-matched baseline to determine whether the improvements arise merely from combining complementary molecular representations. The experiments were performed on the MoleculeNet datasets. Results shown in Figure 3.

**FIGURE 3:**
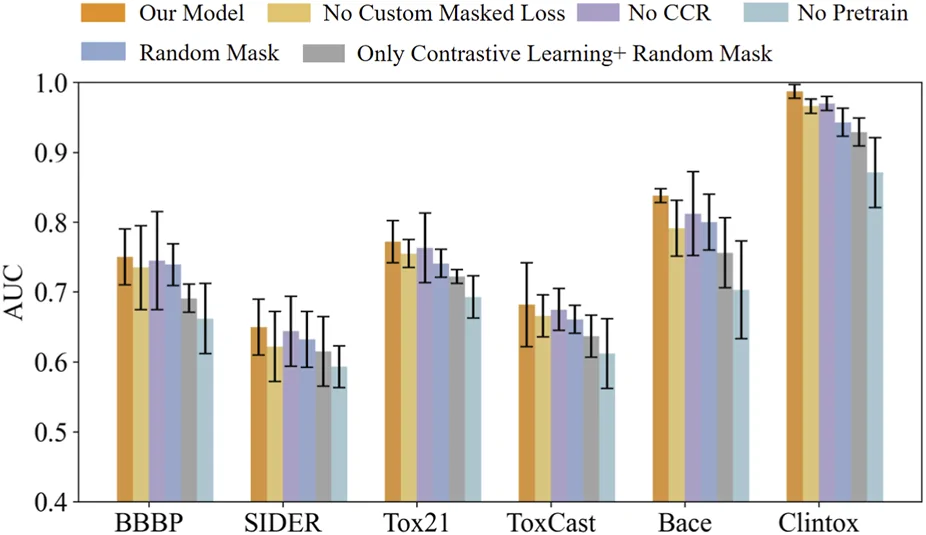
Results of ablation studies on MoleculeNet.

The results demonstrate the contribution of each component to the overall performance. Molrep-SCF achieves the best results across all evaluated datasets. No Pretrain causes the largest performance drop, confirming the importance of self-supervised pretraining. Removing CCR particularly degrades performance on BACE and Tox21, while random masking and the removal of the custom masked loss also reduce predictive accuracy. Notably, the Simple Fusion with Random Mask baseline remains consistently inferior to Molrep-SCF despite using the same modality-specific encoders and combining the same 1D, 2D, and 3D information, although simple feature concatenation has been shown to provide performance benefits (Yang et al., 2019; Liu et al., 2025). These results indicate that the improvements cannot be attributed solely to the aggregation of complementary representations; instead, the proposed alignment, masking, and refinement objectives provide additional benefits.

To assess the robustness of the learned 3D representations, we added mild Gaussian noise to molecular coordinates during both training and testing on QM9. As shown in Table 3, compared with the variant without CCR, Molrep-SCF achieves lower errors on most properties and shows a smaller performance drop under perturbation, indicating that CCR improves the stability and noise resistance of the learned geometric representations.

**TABLE 3:** Impact of CCR on model performance with Gaussian noise added to 3D structures in QM9 dataset.

Methods	Noise added	Alpha	Gap	HOMO	LUMO	Mu	Cv	G298	H298	R2	U298	U0	Zpve	Avg. rank
Molrep-SCF	False	0.053	34.52	23.68	21.47	0.023	0.027	13.03	11.87	0.135	11.98	11.65	1.522	1.0833
No CCR	False	0.059	37.26	23.37	23.43	0.025	0.032	13.88	12.45	0.146	12.09	12.38	1.562	2.167
Molrep-SCF	True	0.072	40.22	24.64	23.66	0.033	0.028	13.93	12.37	0.147	12.38	11.79	1.637	2.833
No CCR	True	0.075	41.78	28.35	24.72	0.029	0.035	14.77	13.46	0.168	13.32	14.32	1.85	3.917

To investigate whether the performance gains arise simply from using multiple conformations or from the topology-guided design of CCR, we compare single-conformation pretraining with different multi-conformation aggregation strategies. As shown in Table 4, multiple conformations generally provide richer geometric supervision than a single conformation. Mean pooling and attention-based aggregation capture part of the complementary information across conformations, but their improvements remain limited because molecular topology is not explicitly considered. In contrast, CCR enables topology-guided interaction and refinement across conformational views, resulting in more consistent and transferable geometric representations.

**TABLE 4:** Comparison of conformer aggregation strategies on QM9. The aggregation strategies are applied during pretraining. Unless otherwise specified, downstream evaluation uses a single conformer, while the parameters learned through CCR during pretraining are retained for fine-tuning.

Method	Conf. Num	Aggregation	Alpha	Gap	HOMO	LUMO	Mu	Cv	G298	H298	R2	U298	U0	ZPVE	Avg. rank
Single	1	None	0.059	37.26	23.37	23.43	0.025	0.032	13.88	12.45	0.146	12.09	12.38	1.562	3.583
Mean	3	Mean pooling	0.060	37.66	23.18	23.19	0.025	0.030	13.43	12.20	0.145	12.31	12.25	1.538	3.000
Attention	3	Attention	0.055	34.78	23.22	21.44	0.025	0.029	13.45	11.96	0.139	12.07	11.98	1.534	2.083
Molrep-SCF	3	Proposed CCR	0.053	34.52	23.68	21.47	0.023	0.027	13.03	11.87	0.135	11.98	11.65	1.522	1.333

### 3.6 Evaluation of other drug-related tasks

For the DTA and CDR evaluations, Molrep-SCF is integrated into the task-specific architectures described in Section 3.1. Specifically, the original drug encoder is replaced with the pretrained Molrep-SCF encoder, while the protein or cell-line encoder, fusion module, prediction head, and optimization settings are kept consistent with the corresponding baseline. This setup ensures that the performance improvements can be primarily attributed to the proposed molecular representation learning approach rather than other architectural changes. The comparative results are presented in Tables 5, 6. The experimental results demonstrate that Molrep-SCF consistently outperforms existing methods on both the DTA and CDR tasks. In comparison to other models, Molrep-SCF shows superior performance in both predictive accuracy and robustness. Notably, its performance on both the Davis and Kiba datasets for DTA, as well as on the PCC and SCC metrics for CDR, indicates its strong ability to capture complex relationships in molecular data. The results suggest that the integration of advanced molecular encoding and pretraining techniques in Molrep-SCF offers a significant advantage, confirming its potential as a highly effective model for drug-related task evaluations.

**TABLE 5:** Performance comparison of Molrep-SCF with baselines on DTA.

Methods	Davis		Kiba	
	CI	MSE	CI	MSE
KronRLS	0.871	0.379	0.782	0.411
SimBoost	0.872	0.282	0.836	0.222
DeepDTA	0.878	0.261	0.863	0.194
WideDTA	0.886	0.262	0.875	0.179
GraphDTA	0.893	0.229	0.882	0.147
DHTNN	0.861	0.319	0.816	0.330
MolPROP	0.852	0.289	0.839	0.278
CSGL	0.874	0.269	0.860	0.203
Molrep-SCF	0.899	0.226	0.893	0.150

**TABLE 6:** Performance comparison of Molrep-SCF with baselines on CDR.

Methods	PCC	SCC
MOLI	81.3	78.2
CDRscan	87.1	85.2
tCNNs	91.0	88.9
DeepCDR	92.3	89.8
DeepTTA	94.1	91.4
DHTNN	86.6	85.2
MolPROP	88.1	87.1
CSGL	90.3	88.5
Molrep-SCF	94.5	92.4

### 3.7 Visualization analysis

To evaluate the quality of molecular embeddings learned by Molrep-SCF, we performed a t-SNE analysis on the Bace dataset to compare the embeddings generated with and without pretraining. Figures 4A,B illustrate the clustering results for pretrained and non-pretrained embeddings. In these visualizations, orange and blue dots represent positive and negative samples. Despite not using Bace labels during the pretraining phase, Molrep-SCF demonstrated strong clustering performance, effectively grouping molecules into distinct categories. Notably, this resulted in a significant improvement in the Davies-Bouldin Index (DBI) (Xiao et al., 2017), a measure of cluster compactness and separation, indicating that pretraining led to more well-defined and discriminative embeddings.

**FIGURE 4:**
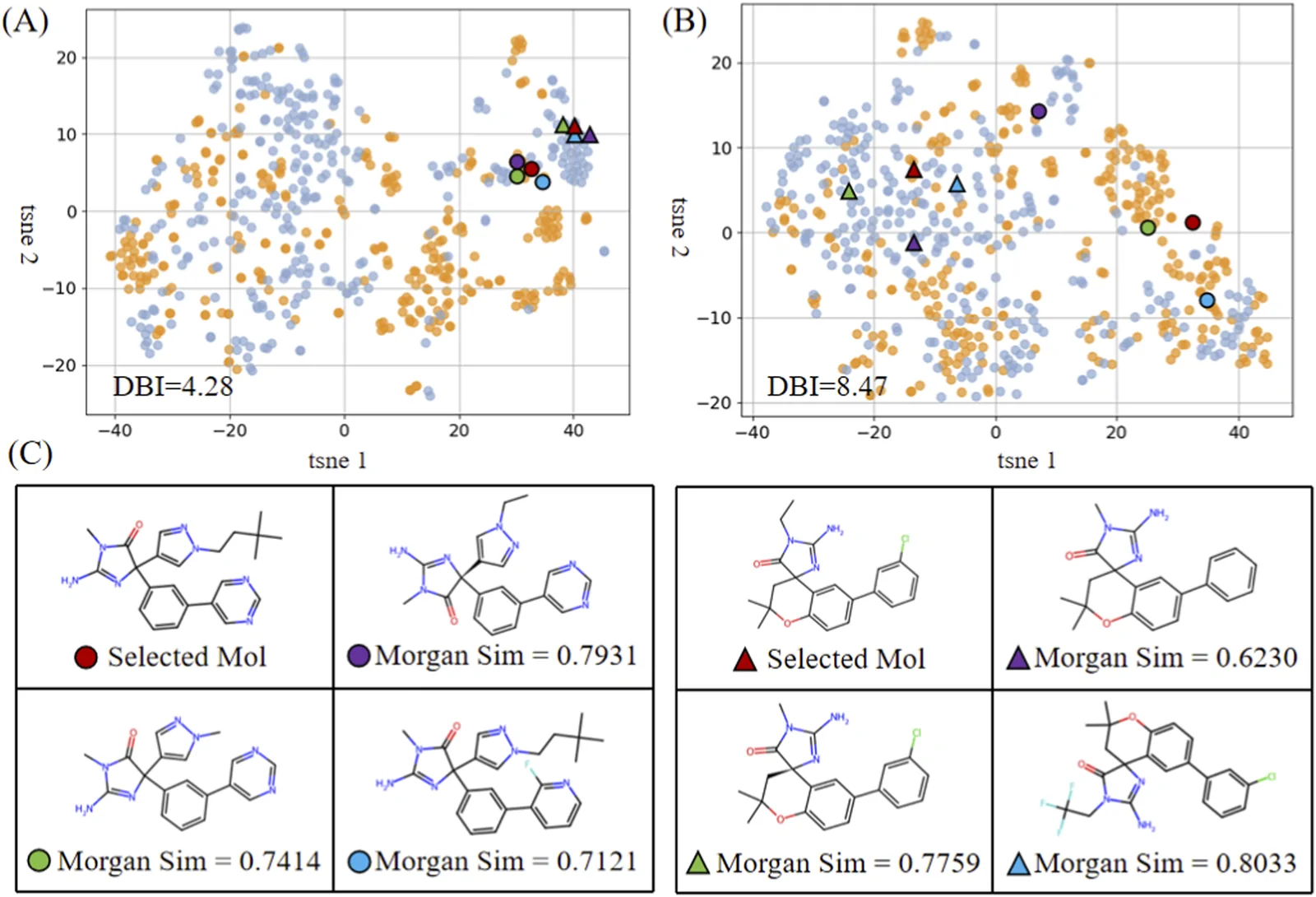
t-SNE visualization of molecular embeddings on BACE dataset. **(A)** Pretrained Molrep-SCF embeddings. **(B)** Non-pretrained Molrep-SCF embeddings. **(C)** Molecular retrieval examples with Morgan similarity scores.

In addition to clustering, we conducted a molecular retrieval task to further investigate the quality of the embeddings. By selecting random query molecules from the Bace dataset, we retrieved structurally similar molecules using cosine similarity on their embedding vectors. The retrieval results were validated using Morgan fingerprints, confirming a high structural similarity between the retrieved molecules (Figure 4C). Pretraining the model further enhanced its retrieval performance, as evidenced by the closer alignment of feature space distributions for structurally similar molecules. This clustering behavior suggests that the model learned more robust and task-relevant representations during pretraining, allowing it to better distinguish between similar and dissimilar molecules. We performed the same analysis on the BBBP dataset (Figure 5).

**FIGURE 5:**
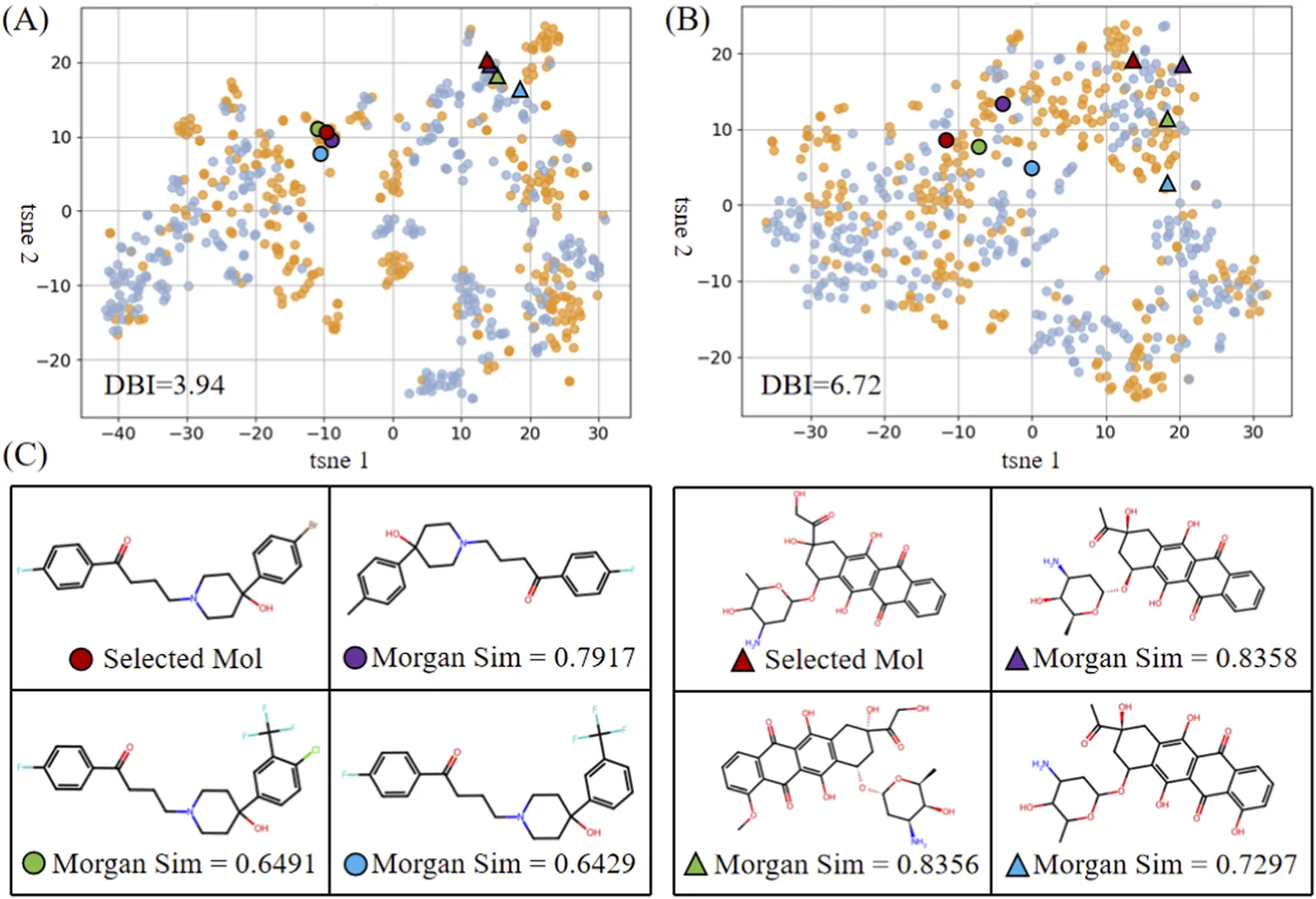
t-SNE visualization of molecular embeddings on BBBP dataset. **(A)** Pretrained Molrep-SCF embeddings. **(B)** Non-pretrained Molrep-SCF embeddings. **(C)** Molecular retrieval examples with Morgan similarity scores.

### 3.8 Attention score

We conducted a visualization study of the attention scores between different modalities, as shown in Figure 6. The red dashed boxes highlight the masked regions in the different modalities. Taking the 1D modality mask token as an example, it is evident that the masked tokens in this modality align well with corresponding regions in the other modalities, where attention scores are notably higher. This suggests that the model is able to learn meaningful correlations between the different representations. Notably, a clear correlation is observed between the 2D and 3D modalities. However, this alignment is not always perfect, especially in cases where the 3D structure exhibits significant conformational folding. In such instances, certain atoms in the 3D structure may be physically closer to the masked region in 1D or 2D, leading to discrepancies in the attention scores.

**FIGURE 6:**
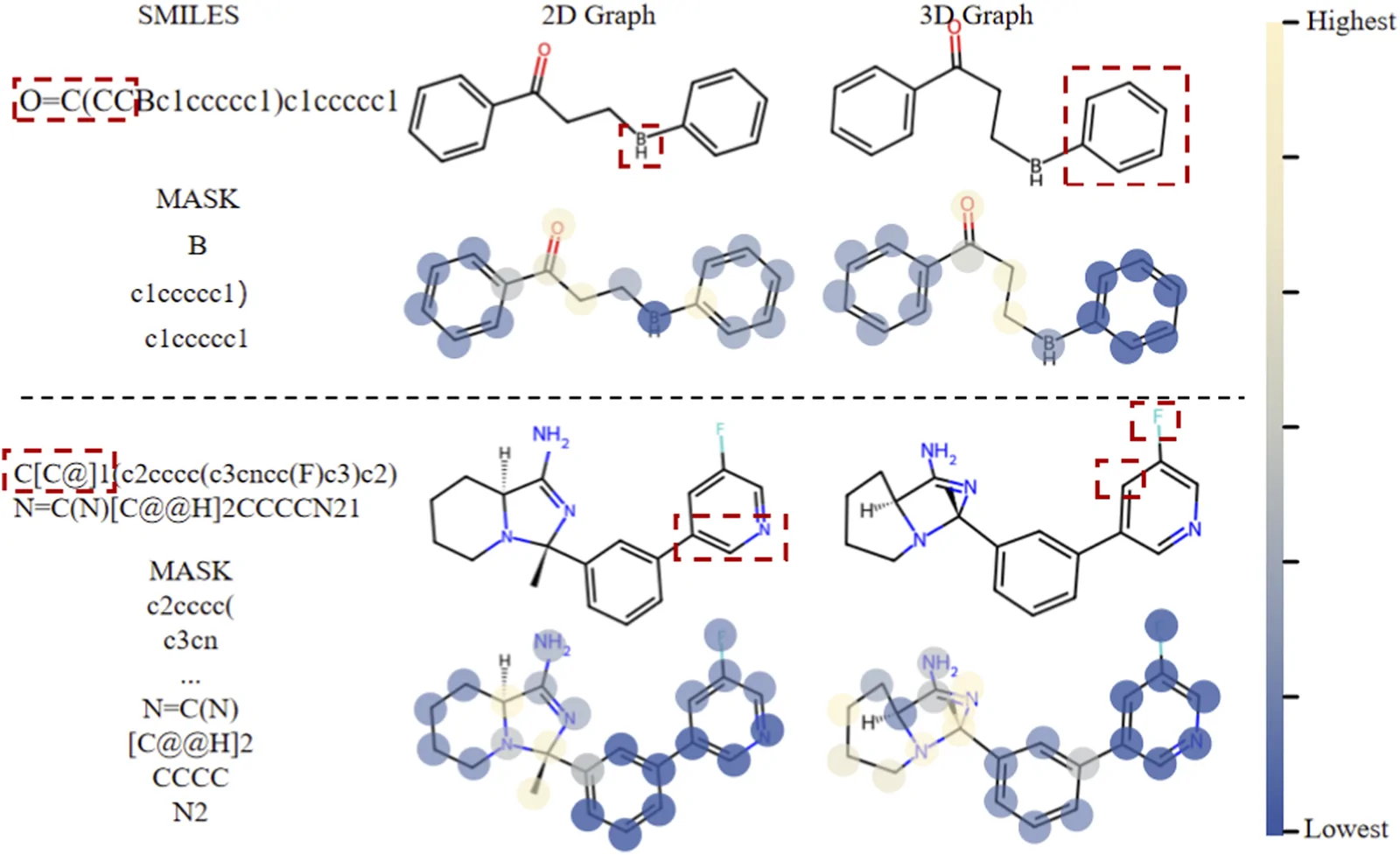
Cross-modal attention visualization across SMILES, 2D graph, and 3D conformations. Red dashed boxes indicate masked tokens or substructures in each modality. The color intensity reflects attention scores, where darker regions denote higher attention weights.

## 4 Conclusion

In conclusion, Molrep-SCF introduces a novel multi-modal framework for molecular representation learning, addressing the challenges of dynamic molecular structures and deep cross-modal semantic relationships. By incorporating a 2D-guided conformational consistency refinement, atomic-level alignment through position-specific masking, and structure-driven cross-modal coupling, Molrep-SCF enhances the consistency and quality of multi-modal molecular representations. These advancements significantly improve performance in molecular property prediction, particularly in scenarios with limited labeled data. Future work will focus on further refining these techniques and exploring their application to more complex molecular tasks.

## Data Availability

The original contributions presented in the study are included in the article/supplementary material, further inquiries can be directed to the corresponding author.
